# Prospective health care costs and lost work days associated with diabetes-related distress and depression symptoms among 1488 individuals with diabetes

**DOI:** 10.1038/s41598-024-52361-4

**Published:** 2024-02-13

**Authors:** Jana Sommer, Ute Linnenkamp, Veronika Gontscharuk, Silke Andrich, Manuela Brüne, Imke Schmitz-Losem, Johannes Kruse, Silvia M. A. A. Evers, Mickaël Hiligsmann, Barbara Hoffmann, Andrea Icks

**Affiliations:** 1 Institute for Health Services Research and Health Economics, German Diabetes Center (DDZ), Düsseldorf at the Heinrich-Heine University Düsseldorf, Leibniz Center for Diabetes Research at the Heinrich Heine University, Auf’m Hennekamp 65, 40225 Duesseldorf, Germany; 2 Institute for Health Services Research and Health Economics, Centre for Health and Society, Medical Faculty, Heinrich Heine University, Moorenstraße 5, 40225 Duesseldorf, Germany; 3 German Center for Diabetes Research (DZD), Ingolstaedter Landstraße 1, 85764 Neuherberg, Germany; 4 Department of Health Services Research, CAPHRI Care and Public Health Research Institute, Maastricht University, Maastricht, The Netherlands; 5 Pronova BKK, Statutory Health Insurance, 67058 Ludwigshafen, Germany; 6 Clinic for Psychosomatic and Psychotherapy, University Clinic Gießen, Friedrichstraße 33, 35392 Gießen, Germany; 7 Trimbos Institute, Netherlands Institute of Mental Health and Addiction, Utrecht, the Netherlands; 8 Institute for Occupational, Social and Environmental Medicine, Centre for Health and Society, Faculty of Medicine, Heinrich-Heine University Düsseldorf, Gurlittstr. 55/II, 40223 Düsseldorf, Germany

**Keywords:** Diabetes complications, Epidemiology

## Abstract

The aim of this study was to investigating the impact of major depression symptoms and diabetes-related distress on future health care costs and lost workdays in individuals with diabetes. We linked survey data from a random sample of a German statutory health insurance (SHI) with diabetes (n = 1488, 63.0% male, mean age 66.9 years) with their SHI data one year after the survey. Within the survey data we identified major depression symptoms (Patient Health Questionnaire-9) and diabetes-related distress (Problem Areas in Diabetes Scale). We retrieved health care costs and lost workdays from SHI data. To assess the impact of major depression symptoms and diabetes-related distress on health care costs and lost workdays, we adjusted regression models for age, sex, education, employment status, and diabetes duration, type, and severity. Major depression symptoms were associated with significantly higher costs (by a factor of 1.49; 95% CI: 1.18–1.88). Lost workdays were also more likely for respondents with depression symptoms (RR1.34; 0.97–1.86). Health care costs (by a factor of 0.81; 0.66–1.01) and the risk of lost workdays (RR 0.86; 0.62–1.18) may be lower among respondents with high diabetes-related distress. While major depression and diabetes-related distress have overlapping indicators, our results indicate different impacts on health care costs.

## Introduction

The number of people affected by diabetes mellitus rose to 463 million adults worldwide in 2019^1^. Health care utilization and costs have been described as about twice as high for individuals with diabetes compared with individuals without diabetes and impose a large economic burden on health care systems globally^2–4^. One important reason for the increased utilization of health care among persons with diabetes is the treatment of diabetes-related late complications. Also, mental comorbidities, such as depression, have been found to be associated with a 1.4-fold up to a 4.5-fold increase in total costs among individuals with diabetes^5,6^. (for summarizing reviews, see also^7,8^) However, most studies have used a cross-sectional design to assess the impact of depression symptoms, and only a few studies have analyzed the association of comorbid depression symptoms prospectively^2,9,10^. For example, Simon et al.^2^ showed that individuals with diabetes and symptoms of depression had 70% higher health care costs in the subsequent six months compared with individuals with diabetes and no symptoms of depression. Moreover, most studies have included only direct medical costs. A few studies also looked at indirect costs, for example, loss in productivity. These studies^11–14^ found that individuals with diabetes and depression symptoms had significantly more lost workdays and an increased loss of productivity, respectively.

However, the mental burden of dealing with diabetes cannot be attributed to depression alone. Beyond this, a concept that stands out clinically and empirically is diabetes-related distress. This term describes an inadequate emotional adjustment to diabetes (e. g., frustration, helplessness)^15,16^ and the worries, concerns, and fears of individuals living with diabetes^17^. A systematic review reported that more than one third of individuals with Type 2 diabetes experience diabetes-related distress^18^. Similar to depression, diabetes-related distress is associated with increased glycosylated hemoglobin A1c (HbA1c) levels, poor disease self-management, negative mental health outcomes, and other worse clinical outcomes^19–22^. Studies have discussed the probability of a mutual relationship between depression and diabetes-related distress. They share signs, such as lethargy, irritability, and weight change, sometimes making it difficult to distinguish between the two conditions^23^. However, recent studies have suggested that diabetes-related distress moderates the association of depression symptoms and lower glycemic control,^16,24–27^ suggesting that depression symptoms and diabetes-related distress are not interchangeable^16^. In the case of health care costs and lost workdays, the impact of diabetes-related distress has not yet been investigated. Given the fact that depression and diabetes-related distress share several symptoms, and depression symptoms are associated with an increase in health care costs and lost workdays, it is therefore of interest whether this association is also found for diabetes-related distress.

Until now, no study has compared the risk of future lost workdays among individuals with diabetes by addressing both depression symptoms and diabetes-related distress. Apart from this, it is also worth knowing whether the results of previous studies on the impact of depression on health care costs and lost workdays, which were mainly carried out in the U. S. (e.g.^9–12^) a country with a primarily private health care market, can be transferred to other countries with health care systems that are based on a government-supported social insurance system, for example, Germany. The aim of this study is thus to examine and compare the impact of depression symptoms and diabetes-related distress on future health care costs and the risk of lost workdays in a large sample of adults with diabetes in Germany.

## Research design and methods

The study design and recruitment of participants have been described in detail elsewhere^28^. We conducted a prospective population-based study. A survey was performed in a random sample of individuals with diabetes insured by the pronova BKK, a statutory health insurance (SHI) in Germany covering 673,366 individuals. The survey data were individually linked to longitudinal SHI data that covered one year after the survey quarter.

### Study population

To identify individuals with diabetes mellitus, we used an algorithm that has been validated and used in previous studies^29^: an insured person was defined as having diabetes if (1) a diagnosis of diabetes mellitus according to the 10th International Classification of Diseases (ICD-10) (E10–E14) was available in at least three of four quarters in 2011, (2) they had at least two prescriptions for antihyperglycemic drugs [Anatomical-Therapeutic-Chemical (ATC) classification A10] in 2011, or (3) they had a single prescription for an antihyperglycemic drug in combination with either a blood glucose or HbA1c measurement or a diagnosis of diabetes in 2011. Out of 46,566 individuals identified as having diabetes in the SHI, 3642 individuals were randomly selected and contacted to participate in the present study (see Suppl Appendix 1 for a flow chart of the recruitment process). The postal recruitment was carried out in March, May and August 2013. Individuals that did not respond within 3–8 weeks, received a reminder letter. Individuals who did not respond to the reminder letter, were contact by telephone 3–7 weeks later at least twice. 1860 people sent back their questionnaire (response rate: 51%) and gave informed consent to use their SHI data^30^. 201 respondents had to be excluded because of missing SHI data during the observation period, e. g., because they switched health insurance. Therefore, 1659 respondents were considered for the analysis, of whom 1490 provided complete information on depression and diabetes-related distress. Two outliers with extremely costly treatments were excluded from the analysis (costs of 103,232 € and 84,852 €). Therefore, data from 1488 respondents were included in the analysis. To analyze lost workdays, we analyzed only employed individuals; thus, 396 individuals were included in the analysis. Ethical approval was obtained from the ethics committee of the medical faculty of the Heinrich Heine University Duesseldorf, Germany, and is available under the study reference 3762. In accordance with the Declaration of Helsinki, participants were informed of the exact procedure of the study and participated voluntarily.

### Measures of depression symptoms and diabetes-related distress

The Patient Health Questionnaire-9 (PHQ-9) provides an effective way to screen for symptoms of major depressive disorder in a primary care setting^31^. It has undergone extensive psychometric testing and is frequently used in populations with diabetes^32,33^. Respondents are asked to indicate how often (“not at all,” “several days,” “more than half the days,” “nearly every day”) nine different symptoms of depression occurred in the past two weeks. Clinically meaningful depression symptoms are defined as five or more of the nine items scored with at least “more than half the days,” and one of these items is “depressed mood” or “anhedonia.” To identify diabetes-related distress, one of the most frequently cited screening tools is the Problem Areas in Diabetes (PAID) scale^34^. The PAID scale is a 20-item scale asking respondents for emotional problems, which are commonly reported among persons with diabetes. Items are answered on a 5-point Likert scale ranging from “no problem” (0) to “a major problem” (4). High diabetes-related distress is assumed for scores of 40 points or more^16^.

### Outcome measures

#### Health care costs in the year after the survey

Health care costs were derived from the SHI data for 1 year after the survey quarter differing for each individual depending on the timing of the survey response. We included all costs for inpatient care, outpatient care, medication and assistive devices, and other health care costs including transportation and travel costs. Over-the-counter (OTC) medications were not included as they are not registered in the SHI systems. Health care costs were calculated from the SHI perspective by using net costs provided by the SHI. Costs were analyzed for every individual for the four quarters that followed the survey quarter.

#### Lost workdays in the year after the survey

An incapacity to work is defined as a condition in which an insured person is not able to work due to medical reasons. In Germany, lost workdays are a result of sick leave taken as a result of a decision made by the treating physician. The number of lost workdays is reported by the employer to the SHI. Lost workdays were analyzed for every employed individual for the four quarters that followed the survey quarter.

### Possible associated variables and confounders

In the survey, we assessed socio-demographic factors, such as age, gender, employment status (yes/no), and education. For education, the International Standard Classification of Education (ISCED) was used to categorize respondents according to their years of education (10 years or less, 11–13 years, 14 years or more)^35^. As disease-specific factors, we assessed the type of diabetes (Type 1, Type 2, other/do not know), duration since the diagnosis of diabetes (in years), and the adapted Diabetes Complications Severity Index (aDCSI)^36^ (based on SHI data from the four quarters before the survey), which assesses the severity of diabetes on the basis of complication development. The aDCSI has been shown to predict hospitalizations, mortality, and health-care-associated costs^37^.

### Statistical analyses

To evaluate whether depression symptoms or diabetes-related distress influence future health care costs and lost workdays, we conducted two-exposure models. To investigate the associations of symptoms of depression (yes/no) and diabetes-related distress (high/not high) with health care costs (continuous), we calculated one generalized linear model (GLM) with a Gaussian distribution (as one person had no health care costs at all we decided to not use gamma distribution) and log link (the latest due to the skewed distribution of health care costs. We adjusted the model for age (continuous), sex (male/female), education (10 years or less, 11–13 years, 14 years or more), employment status (yes/no), duration of diabetes (continuous), type of diabetes (Type 1, Type 2, other/do not know), and diabetes severity (continuous). To identify whether depression symptoms or diabetes-related distress were associated with having lost at least one workday, we used a modified Poisson regression model^38^ for employed individuals with the same independent variables as above, except for employment status. To check whether the effects of exposure were indeed additive, we compared the results of the two-exposure models (depression symptoms and distress) with corresponding single-exposure models with only depression symptoms or diabetes-related distress as one exposure. Moreover, we studied the association between health care costs and diabetes-related distress in more detail by comparing cost percentiles in diabetes-related distress strata. As sensitivity analyses, we computed the same models as above for participants with different cut-offs for cost. For all statistical tests, we considered an α of 0.05 to determine statistically meaningful results. All statistical analyses were performed using the SAS software, V.9.4 (SAS Institute Inc., Cary, NC).

## Results

### Sample characteristics

The final sample of 1488 respondents (37.0% female) had ages that ranged from 18 to 80 years (*M* = 66.9, *SD* = 10.0). As their years of education, 308 respondents indicated 10 or fewer years, 856 indicated 11 to 13 years, and 324 reported 14 or more years. In total, the proportion of respondents with symptoms of major depression was 5.7% (*n* = 85), and the proportion of respondents with diabetes-related distress was 14.1% (*n* = 209; see Table 1). Table 1 presents the clinical and demographic characteristics of the sample.

**Table 1:** Study sample characteristics.

Variable	%/*M/*median (*SD/Q*1–*Q*3)	*n*
Sex		
Female	37.0%	551
Male	63.0%	937
Age		
Mean (*SD*)	66.9 (10.0)	1488
Median (*Q*1–*Q*3)	69.0 (61.0–75.0)	
Years of education		
≤ 10 years	20.7%	308
11–13 years	57.5%	856
≥ 14 years	21.8%	324
Employment status		
Yes	26.6%	396
No	73.4%	1092
Type of diabetes		
Type 1	8.3%	124
Type 2	85.7%	1275
Other/do not know	6.0%	89
Duration of diabetes		
Mean (*SD*)	10.7 (8.2)	1488
Median (*Q*1–*Q*3)	9.0 (5.0–15.0)	
PHQ-9		
Symptoms of major depression	5.7%	85
No symptoms of major depression	94.3%	1403
PAID		
≤ 39 no high diabetes-related distress	85.9%	1279
≥ 40 high diabetes-related distress	14.1%	209
Diabetes severity (aDCSI)		
Mean (*SD*)	2.5 (2.0)	1488
Median (*Q*1–*Q*3)	2.0 (1.0–4.0)	

*M*  means, *SD*  + /− standard deviation, *%* percent.

### Costs and lost workdays

Descriptively, respondents with symptoms of major depression or high diabetes-related distress had higher mean costs and mean lost workdays in the following year than respondents with no symptoms of major depression or without high diabetes-related distress, respectively (see Table 2). Table 2 presents health care costs and lost workdays in relation to depression symptoms and diabetes-related distress.

**Table 2:** Direct health care costs and lost workdays in relation to depression symptoms or diabetes-related distress in the year after the survey.

	PHQ-9			PAID		
	Symptoms of major depression	No symptoms of major depression	*p*-value	High distress (≥ 40)	No high distress (≤ 39)	*p*-value
Health care costs in €						
Mean (*SD*)	7330.2 (10,455.3)	4575.3 (6885.0)	< 0.001	5042.9 (7078.7)	4682.0 (7157.6)	< 0.001
Median (*Q*1–*Q*3)	3996.6 (2004.1–7220.0)	2235.0 (1195.9–4483.1)		3194.3 (1727.6–5805.7)	2199.4 (1180.6–4504.0)	
	n = 85	n = 1403		n = 209	n = 1279	
Lost workdays						
Mean (*SD*)	55.1 (80.4)	17.5 (46.5)	0.004	32.0 (66.2)	17.9 (46.6)	0.252
Median (*Q*1–*Q*3)	29.0 (0.0–78.5)	0.0 (0.0–13.5)		0.0 (0.0–30.0)	0.0 (0.0–15.5)	
	n = 28	n = 368		n = 64	n = 332	

*SD* standard deviation*.*

p-values are based on Mann–Whitney U test.

### Regression analysis on health care costs and lost workdays

**Figure 1:**
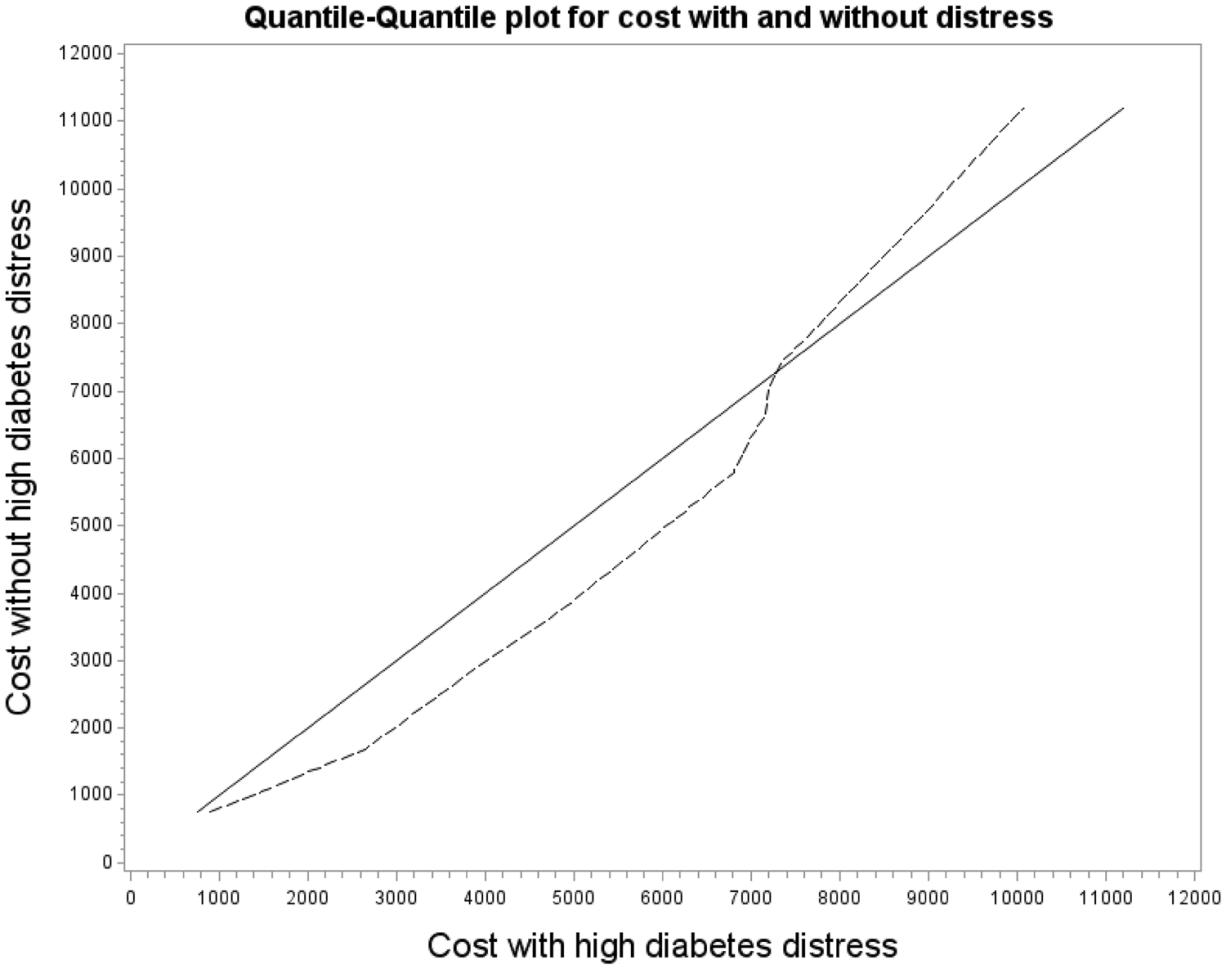
Quantile–quantile plot for cost with and without distress.

We found that respondents with symptoms of major depression had 49% higher health care costs (95% CI 1.18–1.88) compared with respondents without symptoms of major depression in the year after the survey (see Table 3). Respondents experiencing high diabetes-related distress tended to have 19% lower future health care costs (95% CI 0.66–1.01) compared with respondents who did not experience high diabetes-related distress. However, these results were not significant. The corresponding single-exposure models led to very similar estimates and are thus not reported. In the sensitivity analysis, we found that the influence of the PAID scale was reversed for costs below 10,000 € (see Suppl Appendix 2). In the upper range of costs (> 10,000 €), respondents with high diabetes-related distress had lower costs compared with respondents without high diabetes-related distress, while for costs up to 10,000 €, respondents with high diabetes-related distress had higher costs compared with respondents without high diabetes-related distress (see Fig. 1).

**Table 3:** Regression analyses predicting health care costs and lost workdays.

Variables	Mean estimate*	95% CI		*p*-value
**Factors associated with health care costs (n = 1488)**				
Depression symptoms (PHQ-9: major vs. no major depression)	**1.49**	**1.18**	**1.88**	** < 0.001**
Diabetes-related distress (PAID ≥ 40 high distress vs. ≤ 39 no high distress)	0.81	0.66	1.01	0.057
Age (in years)	**0.99**	**0.98**	**1.00**	**0.017**
Sex (female vs. male)	1.07	0.93	1.24	0.325
*Education*				
11–13 years vs. 10 years or less	0.86	0.74	1.01	0.061
14 years or more vs. 10 years or less	0.86	0.70	1.05	0.138
14 years or more vs. 11–13 years	0.99	0.82	1.20	0.943
Employment status (yes vs. no)	**0.67**	**0.54**	**0.84**	** < 0.001**
Duration of diabetes (in years)	**1.01**	**1.00**	**1.02**	**0.012**
*Type of diabetes*				
Type 1 vs. type 2	1.00	0.77	1.30	0.999
Other vs. type 2	0.93	0.67	1.29	0.650
Other vs. type 1	0.93	0.62	1.38	0.707
Diabetes severity (aDCSI)	**1.18**	**1.15**	**1.22**	** < 0.001**
**Factors associated with at least one lost day of work** (n = 396^†^)				
Depression symptoms (PHQ-9: major vs. no major depression)	1.34	0.97	1.86	0.075
Diabetes-related distress (PAID: ≥ 40 high distress vs. ≤ 39 no high distress	0.86	0.62	1.18	0.338
Age (in years)	**0.95**	**0.93**	**0.96**	** < 0.001**
Sex (female vs. male)	0.85	0.67	1.08	0.174
*Education*				
11–13 years vs. 10 years or less	0.82	0.61	1.10	0.185
14 years or more vs. 10 years or less	0.79	0.58	1.07	0.126
14 years or more vs. 11–13 years	0.96	0.76	1.20	0.697
Duration of diabetes (in years)	0.99	0.98	1.01	0.446
*Type of diabetes*				
Type 1 vs. type 2	0.89	0.64	1.24	0.485
Other vs. type 2	0.92	0.60	1.42	0.700
Other vs. type 1	1.03	0.61	1.75	0.904
Diabetes severity (aDCSI)	**1.06**	**1.00**	**1.12**	**0.045**

*Mean estimates are the expected relative mean differences when referring to costs and relative risks when referring to lost workdays.

^†^Employed respondents only.

Significant values are in bold.

Lost workdays were analyzed only among respondents who were employed (n = 396). Respondents with symptoms of major depression tended to be more likely (+ 34%; 95% CI: 0.97–1.86) to have lost at least one day of work in the following year than respondents without symptoms of major depression, but this association was not significant. Among respondents with high diabetes-related distress compared with those without high diabetes-related distress, we found a tendency toward a lower risk of lost workdays (–14%; 95% CI: 0.62–1.18). Similarly, to the results of respondents with symptoms of major depression these results were not significant. The corresponding single-exposure models led to very similar results and are thus not reported. Table 3 presents results from the regression analyses on health care costs and lost workdays.

## Conclusion

Prior work has documented, that individuals with diabetes and depression or psychological distress show a higher utilization of health care (e.g.^7^). However, previous studies have either investigated the associations in cross-sectional designs, focused on the influence of cost as a single factor of health care utilization, or focused on depression symptoms only (e.g.^7,8^). Moreover, no study has investigated the impact of diabetes-related distress on future health care costs and future lost workdays. In our study, we used records from a large German SHI so we could capture participants’ total health care costs and lost workdays for a full year after the survey. Hence, we were able to investigate the factors associated with the future costs of health care and lost workdays in one data set.

As expected, we found that depression symptoms in individuals with diabetes were associated with significantly higher health care costs in the following year. Depression symptoms tended to also predict lost workdays in the following year, but the differences were not significant. In the case of diabetes-related distress, surprisingly, it tended to be associated with lower future health care costs and a lower risk of losing workdays in individuals with diabetes. However, this was the case only in the regression models, and the results were not significant.

Our findings extend results on future health care costs by Simon et al.^2^, Gilmer et al.^10^, Huang et al.^5^, Vamos et al.^14^, and Ciechanowski et al.^9^, in confirming that depression symptoms may act as a significant independent predictor of health care costs. Respondents with symptoms of major depression had almost 50% higher total health care costs compared with respondents without symptoms of major depression in the year after the survey. This is in line with findings from Simon et al.^2^, who found that health care costs were approximately 70% higher for individuals with symptoms of major depression compared with those without any symptoms of depression for the six months after the survey. Additionally, it seems that individuals with diabetes and depression symptoms incur higher costs, irrespective of the health care system in which they are receiving their care. The study by Simon et al.^2^ was carried out in the US, which has a mainly private market, whereas our results were based on data from a statutory health insurance system in Germany.

We found that respondents with high diabetes-related distress tended to have lower future health care costs than respondents without high diabetes-related distress, but this result was not significant. At first glance, this seems somewhat surprising as one could assume that individuals who experience high distress are more likely to be sick and would therefore incur higher health care costs. However, one reason for our finding might be that individuals who are highly engaged in their diabetes self-management experience also have higher diabetes-related distress and—as a consequence—experience fewer short- and long-term complications and produce lower health care costs. Furthermore, they might also feel more burdened by the disease. This would be in line with previous findings, in which comorbidities were shown to be a strong predictor of future health care costs^5^, independent of depression symptoms or distress. Moreover, our results on diabetes-related distress and costs seem to be reversible for higher costs, and therefore, different results are possible, depending on the statistical methods. For instance, we observed 19% lower costs for respondents with high diabetes-related distress in the regression analysis after adjusting for several covariates (see Table 3), even though a non-parametric comparison (see Table 2) showed that respondents with high diabetes-related distress had higher costs. However, in the sensitivity analysis, which included only respondents with costs up to 10,000 €, we found 14% higher costs among those with high diabetes-related distress (see Suppl Appendix 2), a finding that is in line with the results of the non-parametric comparison. In the sensitivity analysis, we found that the risk of lost workdays in the following year in individuals with symptoms of major depression tended to be higher compared with individuals with no symptoms of major depression. This result is in line with previous findings in cross-sectional designs^11,13^. However, for diabetes-related distress, our data point in the opposite direction: high diabetes-related distress tended to predict a lower risk of lost workdays in the following year. Considering the mean age of our sample (66.9 years), one could argue that those who are still working are healthier than those who are not working and are probably more engaged in their diabetes self-management and thus experience higher diabetes-related distress; as a consequence, they have fewer short- and long-term complications, which in turn lead to fewer lost workdays.

### Strengths and limitations

A number of limitations should be considered when interpreting the results. First, only individuals from one SHI could participate in the study, which might influence the results, as the populations insured with the different SHIs in Germany differ considerably^39^. The results are therefore not representative of the German population with diabetes at large. Second, to assess depression symptoms, we used self-report questionnaires; nevertheless, the gold standard for assessing depression is the clinical interview. Therefore, we cannot conclude that all respondents identified with depression symptoms would have been diagnosed with major depression in an interview with a clinician. However, the PHQ-9 provides a standardized measure of the DSM-IV criteria for major depression and has been found to discriminate well between individuals with and without major depression (the area under the curve in a ROC analysis was 0.95 in diagnosing major depression)^31^. The reported prevalence of 5.7% is indeed low however within the range reported by a recent systematic review where prevalence rates among patients with diabetes ranged from 1.8 to 88.6%^40^. The authors found that prevalence estimates based on clinical samples were generally higher than prevalence estimates of community samples possibly due to differences in participant characteristics and a greater severity of depression within clinic settings. Moreover, they found the lowest prevalence based on the PHQ-9 with a cut-off ≥ 10 in a Chinese sample^41^. Thus, our prevalence estimates seem to be low however within the range of expected outcomes. Third, depending on the effect size, the number of employed respondents might not have provided enough power to detect significant differences in the analysis of the risk of lost workdays.

On the other hand, the study has several strengths. The DiaDec study had a reasonably high response rate (51%) for a survey-based study, and the data set was rather large and thus allowed for robust estimates. Even though antihyperglycemic medication, health care utilization, and medication utilization were higher among people who responded to the survey than those who did not, there was no difference between the two groups regarding previous diagnosis of depression^30^. Moreover, our design allowed us to link longitudinal SHI data with the survey data.

### Implications

Combining information about health care costs and lost workdays in one data set provided evidence of the impact of depression symptoms and diabetes-related distress. Many health care plans focus on glycemic control to reduce costs. Our results show that more attention should especially be paid to individuals with diabetes and depression symptoms, as not only their present (e.g.^7,8,11,42^) but also their future costs and losses in productivity are major issues. A successful treatment of depression among individuals with diabetes should be a major goal in disease management programs; these may include, for example, psychotherapy (individual, group-based or online), pharmacological treatment, improved diabetes self-management, regular physical activity, or stress reduction techniques^43–45^. This is of high clinical relevance and could contribute to a massive reduction in health care costs and is thus of great importance to health care professionals and policy makers. However, further research is necessary to demonstrate whether these results hold true in other samples as well.

In summary, our results for depression symptoms and diabetes-related distress point in opposite directions. Although the concepts of depression and diabetes-related distress may overlap to some extent, our results indicate that, in the case of health care costs and lost workdays, they probably do not.

To our knowledge, this is the first study to compare future health care costs and lost workdays for individuals with diabetes reporting major depression symptoms or high diabetes-related distress. Further studies are needed to confirm whether our findings hold true in other samples as well.

### Supplementary Information

Supplementary Information 1.

Supplementary Information 2.

## Data Availability

The data that support the findings of this study is not publicly available. Data can be shown by the corresponding authors at the Institute upon reasonable request.

## References

[1] Saeedi P, Petersohn I, Salpea P (2019). Global and regional diabetes prevalence estimates for 2019 and projections for 2030 and 2045: Results from the International Diabetes Federation Diabetes Atlas, 9th edition. Diabetes Res. Clin. Pract..

[2] Simon GE, Katon WJ, Lin EH (2005). Diabetes complications and depression as predictors of health service costs. Gen. Hosp. Psychiatry.

[3] Williams R, Karuranga S, Malanda B (2020). Global and regional estimates and projections of diabetes-related health expenditure: Results from the International Diabetes Federation Diabetes Atlas, 9th edition. Diabetes Res. Clin. Pract..

[4] Janssen LMM, Hiligsmann M, Elissen AMJ (2020). Burden of disease of type 2 diabetes mellitus: Cost of illness and quality of life estimated using the Maastricht Study. Diabet. Med..

[5] Huang CJ, Hsieh HM, Chiu HC (2017). Health care utilization and expenditures of patients with diabetes comorbid with depression disorder: A national population-based cohort study. Psychiatry Invest..

[6] Brüne M, Linnenkamp U, Andrich S (2021). Health care use and costs in individuals with diabetes with and without comorbid depression in Germany: Results of the cross-sectional DiaDec study. Diabetes Care.

[7] Hutter N, Schnurr A, Baumeister H (2010). Health care costs in patients with diabetes mellitus and comorbid mental disorders—A systematic review. Diabetologia.

[8] Molosankwe I, Patel A, José Gagliardino J, Knapp M, McDaid D (2012). Economic aspects of the association between diabetes and depression: A systematic review. J. Affect. Disord..

[9] Ciechanowski PS, Katon WJ, Russo JE (2000). Depression and diabetes: Impact of depressive symptoms on adherence, function, and costs. Arch. Intern. Med..

[10] Gilmer TP, O’Connor PJ, Rush WA (2005). Predictors of health care costs in adults with diabetes. Diabetes Care.

[11] Egede LE, Zheng D, Simpson K (2002). Comorbid depression is associated with increased health care use and expenditures in individuals. Diabetes Care.

[12] Egede LE (2004). Effects of depression on work loss and disability bed days in individuals with diabetes. Diabetes Care.

[13] Subramaniam M, Sum CF, Pek E (2009). Comorbid depression and increased health care utilisation in individuals with diabetes. Gen. Hosp. Psychiatry.

[14] Vamos EP, Mucsi I, Keszei A, Kopp MS, Novak M (2009). Comorbid depression is associated with increased health care utilization and lost productivity in persons with diabetes : A large nationally representative Hungarian population survey. Psychosom. Med..

[15] Gonzalez JS, Fisher L, Polonsky WH (2011). Depression in diabetes: Have we been missing something important?. Diabetes Care.

[16] Snoek FJ, Bremmer MA, Hermanns N (2015). Constructs of depression and distress in diabetes: Time for an appraisal. Lancet Diabetes Endocrinol..

[17] Polonsky WH, Fisher L, Earles J (2005). Assessing psychosocial distress in diabetes: Development of the diabetes distress scale. Diabetes Care.

[18] Perrin NE, Davies MJ, Robertson N, Snoek FJ, Khunti K (2017). The prevalence of diabetes-specific emotional distress in people with Type 2 diabetes: A systematic review and meta-analysis. Diabet. Med..

[19] Fisher L, Glasgow RE, Strycker LA (2010). The relationship between diabetes distress and clinical depression with glycemic control among patients with type 2 diabetes. Diabetes Care.

[20] Reddy J, Wilhelm K, Campbell L (2013). Putting PAID to diabetes-related distress: The potential utility of the problem areas in diabetes (PAID) scale in patients with diabetes. Psychosomatics.

[21] De Groot M, Anderson R, Freedland KE, Clouse RE, Lustman PJ (2001). Association of depression and diabetes complications: A meta-analysis. Psychosom. Med..

[22] Schmitt A, Bendig E, Baumeister H, Hermanns N, Kulzer B (2021). Associations of depression and diabetes distress with self-management behavior and glycemic control. Health Psychol..

[23] Holt RI, de Groot M, Lucki I (2014). NIDDK international conference report on diabetes and depression: Current understanding and future directions. Diabetes Care.

[24] Fisher L, Mullan JT, Arean P (2010). Diabetes distress and not clinical depression or depressive affect is associated with glycemic control in both cross-sectional and longitudinal analyses. Diabetes Care.

[25] Fisher L, Polonsky WH, Hessler D (2019). Addressing diabetes distress in clinical care: A practical guide. Diabet. Med..

[26] van Bastelaar KM, Pouwer F, Geelhoed-Duijvestijn PH (2010). Diabetes-specific emotional distress mediates the association between depressive symptoms and glycaemic control in Type 1 and Type 2 diabetes. Diabet. Med..

[27] Schmitt A, Reimer A, Kulzer B (2015). Negative association between depression and diabetes control only when accompanied by diabetes-specific distress. J. Behav. Med..

[28] Kvitkina T, Brüne M, Chernyak N (2016). Protocol of the DiaDec-study: Quality of life, health care utilisation and costs in patients with diabetes: The role of depression. J. Diabetol. Endocrinol..

[29] Icks A, Haastert B, Trautner C (2009). Incidence of lower-limb amputations in the diabetic compared to the non-diabetic population: Findings from Nationwide Insurance Data, Germany, 2005–2007. Exp. Clin. Endocrinol. Diabetes.

[30] Linnenkamp U, Gontscharuk V, Brüne M (2020). Using statutory health insurance data to evaluate non-response in a cross-sectional study on depression among patients with diabetes in Germany. Int. J. Epidemiol..

[31] Kroenke K, Spitzer RL, Williams JB (2001). The PHQ-9: Validity of a brief depression severity measure. J. Gen. Intern. Med..

[32] Van Dijk SEM, Adriaanse MC, van der Zwaan L (2018). Measurement properties of depression questionnaires in patients with diabetes: A systematic review. Qual. Life Res..

[33] Harding KA, Pushpanathan ME, Whitworth SR (2019). Depression prevalence in type 2 diabetes is not related to diabetes–depression symptom overlap but is related to symptom dimensions within patient self-report measures: A meta-analysis. Diabet. Med..

[34] Polonsky WH, Anderson BJ, Lohrer PA (1995). Assessment of diabetes-related distress. Diabetes Care.

[35] *OECD Classifying Educational Programmes Manual for ISCED-97 Implementation in OECD Countries* (article online). http://www.oecd.org/education/skills-beyond-school/1962350.pdf. Accessed 17 June 2022 (1999).

[36] Chang HY, Weiner JP, Richards TM, Bleich SN, Segal J (2021). Validating the adapted diabetes complications severity index in claims data. Am. J. Manag. Care.

[37] Wicke FS, Glushan A, Schubert I (2019). Performance of the adapted Diabetes Complications Severity Index translated to ICD-10. Am. J. Manag. Care.

[38] Zou G (2004). A modified poisson regression approach to prospective studies with binary data. Am. J. Epidemiol..

[39] Hoffmann F, Icks A (2012). Diabetes, “epidemic” in Germany? A critical look at health insurance data sources. Exp. Clin. Endocrinol. Diabetes.

[40] Harding KA, Pushpanathan ME, Whitworth SR, Nanthakumar S, Bucks RS, Skinner TC (2019). Depression prevalence in type 2 diabetes is not related to diabetes-depression symptom overlap but is related to symptom dimensions within patient self-report measures: A meta-analysis. Diabet. Med..

[41] Sun JC, Xu M, Lu JL, Bi YF, Mu YM, Zhao JJ (2015). Associations of depression with impaired glucose regulation, newly diagnosed diabetes and previously diagnosed diabetes in Chinese adults. Diabet. Med..

[42] Von Korff M, Katon W, Lin EHB (2005). Work disability among individuals with diabetes. Diabetes Care.

[43] van der Feltz-Cornelis C, Allen SF, Holt RIG, Roberts R, Nouwen A, Sartorius N (2021). Treatment for comorbid depressive disorder or subthreshold depression in diabetes mellitus: Systematic review and meta-analysis. Brain Behav..

[44] Baumeister H, Hutter N, Bengel J (2014). Psychological and pharmacological interventions for depression in patients with diabetes mellitus: An abridged Cochrane review. Diabet. Med..

[45] Winkley K, Upsher R, Stahl D, Pollard D, Kasera A, Brennan A, Heller S, Ismail K (2020). Psychological interventions to improve self-management of type 1 and type 2 diabetes: A systematic review. Health Technol. Assess..

