# Supplementary figures and images for: Prospective health care costs and lost work days associated with diabetes-related distress and depression symptoms among 1488 individuals with diabetes
Source: Sci Rep. 2024 Feb 13;14:3621. doi: 10.1038/s41598-024-52361-4 (PMC10864264; doi:10.1038/s41598-024-52361-4)

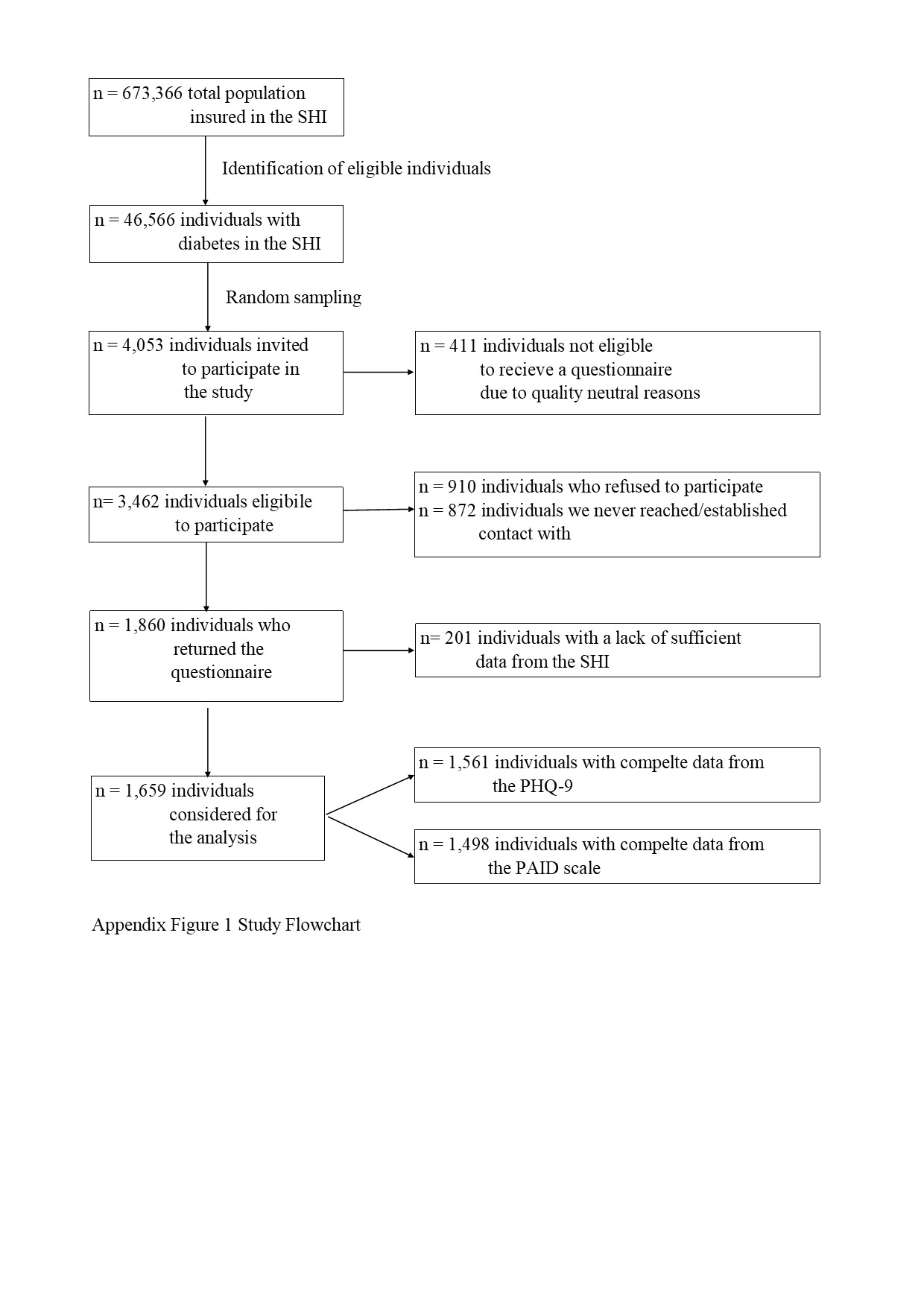

Supplement: Supplementary file 1 — Supplementary Information 1. [file 41598_2024_52361_MOESM1_ESM.jpg]
