# Supplementary material for: Prospective health care costs and lost work days associated with diabetes-related distress and depression symptoms among 1488 individuals with diabetes
Source: Sci Rep. 2024 Feb 13;14:3621. doi: 10.1038/s41598-024-52361-4 (PMC10864264; doi:10.1038/s41598-024-52361-4)
Supplement: Supplementary file 2 — Supplementary Information 2. [file 41598_2024_52361_MOESM2_ESM.docx]

| ***Variables*** | Mean eastimate* | 95 %-CI | | *p*-value |
| --- | --- | --- | --- | --- |
| ***Factors associated with health care costs (n=1322)*** | | | | |
| depression symptoms (PHQ-9 major vs. no major depression) | **1.26** | **1.09** | **1.46** | **0.0019** |
| diabetes-related distress (PAID: ≥40 high distress vs. ≤39 no high distress | **1.14** | **1.03** | **1.27** | **0.0147** |
| age (in years) | 1.00 | 0.99 | 1.00 | 0.9532 |
| sex (female vs. male) | 1.06 | 0.98 | 1.16 | 0.1401 |
| *education* |  |  |  |  |
| 11-13 years vs. 10 years or less | 0.94 | 0.86 | 1.03 | 0.1979 |
| 14 years or more vs. 10 years or less | 0.94 | 0.83 | 1.06 | 0.3264 |
| 14 years or more vs. 11-13 years | 1.00 | 0.90 | 1.11 | 0.9990 |
| employment status (yes vs. no) | 0.93 | 0.84 | 1.04 | 0.2195 |
| duration of diabetes (in years) | **1.01** | **1.00** | **1.01** | **0.0002** |
| *type of diabetes* |  | | | |
| Type 1 vs. Type 2 | 1.00 | 0.86 | 1.16 | 0.9800 |
| other vs. Type 2 | 0.93 | 0.79 | 1.11 | 0.4454 |
| other vs. Type 1 | 0.93 | 0.75 | 1.16 | 0.5282 |
| diabetes severity (aDCSI) | **1.09** | **1.07** | **1.11** | **<0.0001** |
| ***Factors associated with lost workdays (n=374***†***)*** | | | | |
| depression symptoms (PHQ-9 major vs. no major depression) | **1.48** | **1.03** | **2.12** | **0.0348** |
| diabetes-related distress (PAID: ≥40 high distress vs. ≤39 no high distress | 0.86 | 0.62 | 1.21 | 0.3950 |
| age (in years) | **0.94** | **0.93** | **0.96** | **<0.0001** |
| sex (female vs. male) | 0.89 | 0.69 | 1.14 | 0.3502 |
| *education* |  | | | |
| 11-13 years vs. 10 years or less | 0.84 | 0.62 | 1.15 | 0.2823 |
| 14 years or more vs. 10 years or less | 0.81 | 0.58 | 1.13 | 0.2192 |
| 14 years or more vs. 11-13 years | 0.97 | 0.76 | 1.22 | 0.7736 |
| duration of diabetes (in years) | 0.99 | 0.97 | 1.01 | 0.3651 |
| *type of diabetes* |  | | | |
| Type 1 vs. Type 2 | 0.87 | 0.61 | 1.23 | 0.4252 |
| other vs. Type 2 | 0.91 | 0.59 | 1.40 | 0.6585 |
| other vs. Type 1 | 1.04 | 0.61 | 1.80 | 0.8777 |
| diabetes severity (aDCSI) | **1.07** | **1.00** | **1.124** | **0.0487** |
|  | | | | |

**Appendix 2** Regression analyses predicting health care costs and lost workdays with a cut off at 10,000 €

*mean estimates are the expected relative mean differences when referring to costs and relative risks when referring to lost workdays

† employed respondents only
